# PADI2 exacerbates* Mycoplasma ovipneumoniae-*induced lung injury in sheep by suppressing M2 macrophage polarization

**DOI:** 10.1186/s13567-025-01632-7

**Published:** 2025-10-27

**Authors:** Chenbo Yan, Tianning Dong, Chong Zhang, Maojun Liu, Yanli Zhang

**Affiliations:** 1 Jiangsu Livestock Embryo Engineering Laboratory, Nanjing Agricultural University, Nanjing, 210095 China; 2 Key Laboratory of Veterinary Biological Engineering and Technology of Ministry of Agriculture, Institute of Veterinary Medicine, Jiangsu Academy of Agricultural Sciences, Nanjing, 210014 China

**Keywords:** *Mycoplasma ovipneumoniae*, PADI2, macrophage polarization

## Abstract

Macrophages are considered the first line of defence against *Mycoplasma pneumoniae* infection in sheep, and their polarization significantly affects lung tissue damage and remodelling. Here, we collected sheep bronchoalveolar lavage fluid (BALF) 28 days after infection with *Mycoplasma ovipneumoniae* (MO). RNA-seq analysis revealed a significant increase in peptidyl arginine deiminase 2 (PADI2) expression in the MO group, and upregulated genes were significantly enriched in the “FoxO signalling pathway” and “AMPK signalling pathway”. Immunohistofluorescence (IHF) staining revealed a significant increase in the number of macrophages, which were widely distributed in the alveolar cavity, in the MO infection group, and PADI2 was also positively expressed in the macrophages. In bone marrow-derived macrophages (BMDMs) isolated in vitro, inhibiting the expression of PADI2 induced infected macrophages to polarize towards alternatively activated (AAM, M2) macrophages. Moreover, the expression levels of the anti-inflammatory gene IL10 and tissue repair-related genes such as vascular endothelial growth factor alpha (VEGFA), transforming growth factor-β (TGF-β), and hepatocyte growth factor (HGF) significantly increased. In an in vitro coculture system, inhibition of PADI2 expression in BMDMs reduced apoptotic and intracellular reactive oxygen species (ROS) levels in lung fibroblasts. Interestingly, correlation analysis between the tracheal MO load and PADI2 expression in the bronchoalveolar lavage fluid of naturally infected MO sheep revealed that the higher the MO load was, the greater the PADI2 expression. A dual luciferase reporter assay confirmed that this process may be regulated by the transcription factor SP3. These results contribute to the understanding of the polarization state of macrophages after mycoplasma infection and to the preliminary exploration of PADI2 as an effective target to enhance host defence against MO infection. Targeting PADI2 to promote the polarization of M2 macrophages provides a promising approach for treating MO infection or preparing anti-Mycoplasma pneumonia gene-edited sheep.

**Supplementary Information:**

The online version contains supplementary material available at 10.1186/s13567-025-01632-7.

## Introduction

Ovine mycoplasmal pneumonia caused by *Mycoplasma ovipneumoniae* (MO) is a chronic respiratory infectious disease prevalent in global sheep farming regions, particularly in intensive breeding systems [1]. Transmitted via aerosols, MO infection triggers persistent coughing, progressive emaciation, and interstitial pneumonia in lambs and adult sheep, often leading to secondary bacterial infections and mortality, thereby imposing significant economic losses on the livestock industry [2]. Although antibiotic therapies and vaccine development have partially mitigated disease spread, the antigenic variability of MO and its immune evasion mechanisms continue to limit the efficacy of current prevention strategies [3]. Consequently, elucidating the pathogenic mechanisms of MO and its interactions with host immune systems has emerged as a critical research priority.

Recent studies suggest that MO infection remodels the local inflammatory microenvironment by modulating host epigenetic modifications or critical signalling pathways; however, its molecular targets and regulatory networks remain incompletely elucidated. The role of PADI2, a central enzyme in protein citrullination, has garnered increasing attention for its role in inflammatory diseases and infection immunity. The frequent wild introgression of PADI2-specific areas from wild sheep to domestic sheep enhances their climate adaptability and resistance to pneumonia [4]. PADI2 catalyses the conversion of arginine residues to citrulline, modifying target proteins such as histones, chemokines, and antimicrobial peptides and thereby regulating pathological processes, including NF-κB activation and cytokine storms [5]. Notably, previous studies have revealed that the citrullination induced by PADI2 impairs the antibacterial activity of the body against *Staphylococcus aureus*, *Streptococcus pneumoniae*, and nontypable *Haemophilus influenzae* [6]. These findings indicate that the changes in the expression of PADI2 during MO infection and its regulatory mechanism in the host defence response urgently need to be explored.

In this study, we performed RNA-seq on the BALF of sheep infected with MO and reported that PADI2 expression significantly increased after MO infection. We also revealed that inhibiting PADI2 expression can induce the polarization of MO-infected macrophages towards M2 macrophages. In addition, we observed a positive correlation between PADI2 expression and MO loading, and MO-induced PADI2 expression may be regulated by SP3 transcription factors.

## Materials and methods

### *Mycoplasma pneumoniae* disease model and sample collection

As described previously [7], we injected the NJ01 strain (Collection Number: CCTCC NO: M 2020907) of *Mycoplasma pneumoniae* into the trachea of Hu sheep for 29 days to establish a disease infection model. After slaughter, we collected the BALF and divided the samples into two groups (the control group and the MO group) for RNA isolation. Total RNA was extracted and isolated using a TRIzol reagent kit (Tsingke, China) according to the manufacturer’s instructions.

### Transcriptome sequencing

After total RNA was extracted and the quality inspected, poly(A) + RNA enrichment was performed using oligo(dT)-coupled magnetic beads (Thermo Fisher Scientific, USA). Enriched mRNA underwent magnesium-catalysed fragmentation to generate 150–300 nt fragments, which were reverse transcribed into cDNA using the NEBNext Ultra RNA Library Prep Kit for Illumina (New England Biolabs, MA, USA). Library purification involved dual-size selection using AMPure XP beads (Beckman Coulter, USA). Polymerase chain reaction (PCR) amplification was performed with Phusion High-Fidelity DNA Polymerase. The resulting cDNA libraries were subsequently sequenced using an Illumina NovaSeq 6000 by Personal Biotechnology Co., Ltd. (Nanjing, China).

### Bioinformatics analysis

Raw sequencing reads were processed through a standardized bioinformatics pipeline. Initially, adapter-contaminated and low-quality bases (Q < 20) were trimmed using fastp (v0.23.2) with default parameters. Processed reads were then aligned to the Ovis aries reference genome using HISAT2 (v2.2.1) with strand-specific parameters. Transcript assembly was performed using StringTie (v2.2.1) in de novo mode, integrating splice junction information. Gene-level quantification was calculated as transcripts per million (TPM) using RSEM (v1.3.3), incorporating transcript length normalization and multiread correction. We used DESeq to perform differential gene expression analysis and screened genes whose expression was |log_2_FoldChange|> 1 and whose *P* value was < 0.05. Gene Ontology (GO) enrichment and Kyoto Encyclopedia of Genes and Genomes (KEGG) pathway enrichment analyses of the DEGs were performed using R clusterProfiler (v4.8.2).

### IHF staining

After the lungs of the sheep were dissected and extracted, half of the entire lung apex was fixed and embedded for IHF staining using a previously described method [8]. Antibodies against CD68 (28,058–1-AP) and PADI2 (12,110–1-AP) were purchased from Proteintech Group, Inc. (Wuhan, China). Nuclei were stained with DAPI, and images were captured using an LSM 710 laser scanning confocal microscope (Carl Zeiss, Oberkochen, Germany).

### Clinical sample collection and pathological scoring

In March 2025, a commercial sheep farm in Jiangsu Province experienced an outbreak of respiratory diseases. To characterise the pathogen, 24 affected sheep were randomly selected for slaughter. Autopsy involved the collection of lung tissue, BALF and tracheal swab samples. The samples were submitted to an independent third-party diagnostic laboratory for microbiological testing, and only MO was detected.

### Cell culture, cell transfection and MO infection

BMDMs were obtained by flushing the femoral and tibial bone marrow into a 50 mL centrifuge tube with sterile PBS. After centrifugation at 300 × *g* for 5 min, the red blood cells were lysed, and then centrifugation was performed again at 300 × *g* for 5 min. The obtained cells were resuspended at a density of 1–2 × 10⁶ cells/mL in RPMI 1640 medium containing 20% foetal bovine serum (FBS), 1% penicillin–streptomycin solution, and 50 ng of macrophage colony-stimulating factor (M-CSF). The cells were cultured for 7 days, and half of the culture medium was replaced on the third day.

For the isolation and culture of sheep lung fibroblasts, primary lung fibroblasts were isolated from lamb lung tissue using enzymatic digestion as previously described [9], and immunofluorescence staining was used to identify the expression of vimentin and cytokeratins (Additional file 1).

Lipofectamine 3000 transfection reagent was used to transfect pEGFP-N1 (vector) plasmids, pEGFP-N1-PADI2 (PADI2) plasmids, and siRNAs targeting PADI2 (siPADI2) or siNC according to the manufacturer’s instructions. After 12 h of transfection, the cells were inoculated with MO (multiplicity of infection, MOI = 1). The siRNA sequences of PADI2 are listed in Additional file 2.

### Assessment of MO load

To detect the MO load in the trachea, MO qPCR was used to detect the MO DNA. In accordance with the manufacturer’s protocol, we used a magnetic DNA extraction kit (TIANGEN, China) to extract total MO genomic DNA from all the samples. As described previously [7], the MO copy number was determined by qPCR amplification of the MO *tuf* gene. Amplification was performed using a QuantStudio 6 Pro real-time PCR system (Applied Biosystems, Carlsbad, CA, USA).

### Reverse transcription quantitative PCR (RT‒qPCR)

Total RNA was extracted from all the samples using TRIzol. cDNA was synthesized using the HiScript III RT SuperMix for qPCR kit (Vazyme, Nanjing, China). RT‒qPCR was performed on a Bio-Rad iQ5 system (Bio-Rad iQ5 System, Shanghai, China) with a ChamQ Universal SYBR qPCR Master Mix kit (Vazyme, Nanjing, China). The 2^−ΔΔCt^ method was used to normalize mRNA expression levels to those of sheep TUBB to obtain mRNA arbitrary units (fold change). The primers used are listed in Additional file 3.

### Western blotting

BMDMs were collected, and intracellular proteins were detected using a previously described method [10]. Antibodies against TUBB (β-tubulin, 10,094–1-AP) and PADI2 (12,110–1-AP) were purchased from Proteintech Group, Inc. (Wuhan, China).

### Transwell cell coculture

In previous studies, we reported that the number of fibroblasts in lung tissues decreased after MO infection. Therefore, we isolated lung fibroblasts in vitro and cocultured them with BMDMs (Figure 4A). Fibroblasts were digested at 70–80% confluency, centrifuged, resuspended, and seeded into the lower chamber. BMDMs were seeded into the upper chamber with a pore size of 0.4 μm. After the BMDMs were transfected for 12 h, they were inoculated with MO. The coculture system was subsequently placed in an incubator at 37 °C with 5% CO₂ for cultivation. After 24 h, the fibroblasts were collected for flow cytometry analysis, and total RNA was harvested from the BMDMs.

### Flow cytometry

The cells were washed three times with PBS and centrifuged at 500 × *g* for 5 min. The cells were subsequently resuspended in 500 μL of PBS. To investigate the expression of intracellular markers of macrophage activation, the following staining steps were performed. The cells were stained for CD86 (eBioscience 62–0862-82; Thermo Fisher Scientific) and CD206 (eBioscience 17–2061-82; Thermo Fisher Scientific) using flow cytometry staining with an intracellular antigen kit (eBioscience 00–5521-00; Thermo Fisher Scientific) according to the user guidelines provided by Thermo Fisher Scientific.

To measure the ROS generated in lung fibroblasts, a DCFH-DA fluorescent probe solution was mixed with the cells, and the reaction was then performed according to the protocol of the Reactive Oxygen Species Assay Kit (Beyotime, S0033S). Flow cytometry was used to evaluate the ROS levels.

To detect apoptosis, an Annexin V-FITC/PI solution was mixed with the cells, after which the reaction was performed in accordance with the product protocol (BD Biosciences Pharmingen, CA, USA). Flow cytometry was used to determine the ratio of the number of FITC-stained cells to the total number of cells.

### Statistical analysis

Statistical analysis was performed using SPSS Statistics 26 and GraphPad Prism 7.0 (GraphPad Software, San Diego, CA, USA). The data are expressed as the mean ± standard error of the mean (SEM). The results were compared using Student’s *t* test (two-tailed) or one-way ANOVA and the post hoc Tukey test to identify specific differences between groups. Statistical significance was confirmed at a *P* value < 0.05.

## Results

### Identification and enrichment analysis of differentially expressed genes

**Figure 1:**
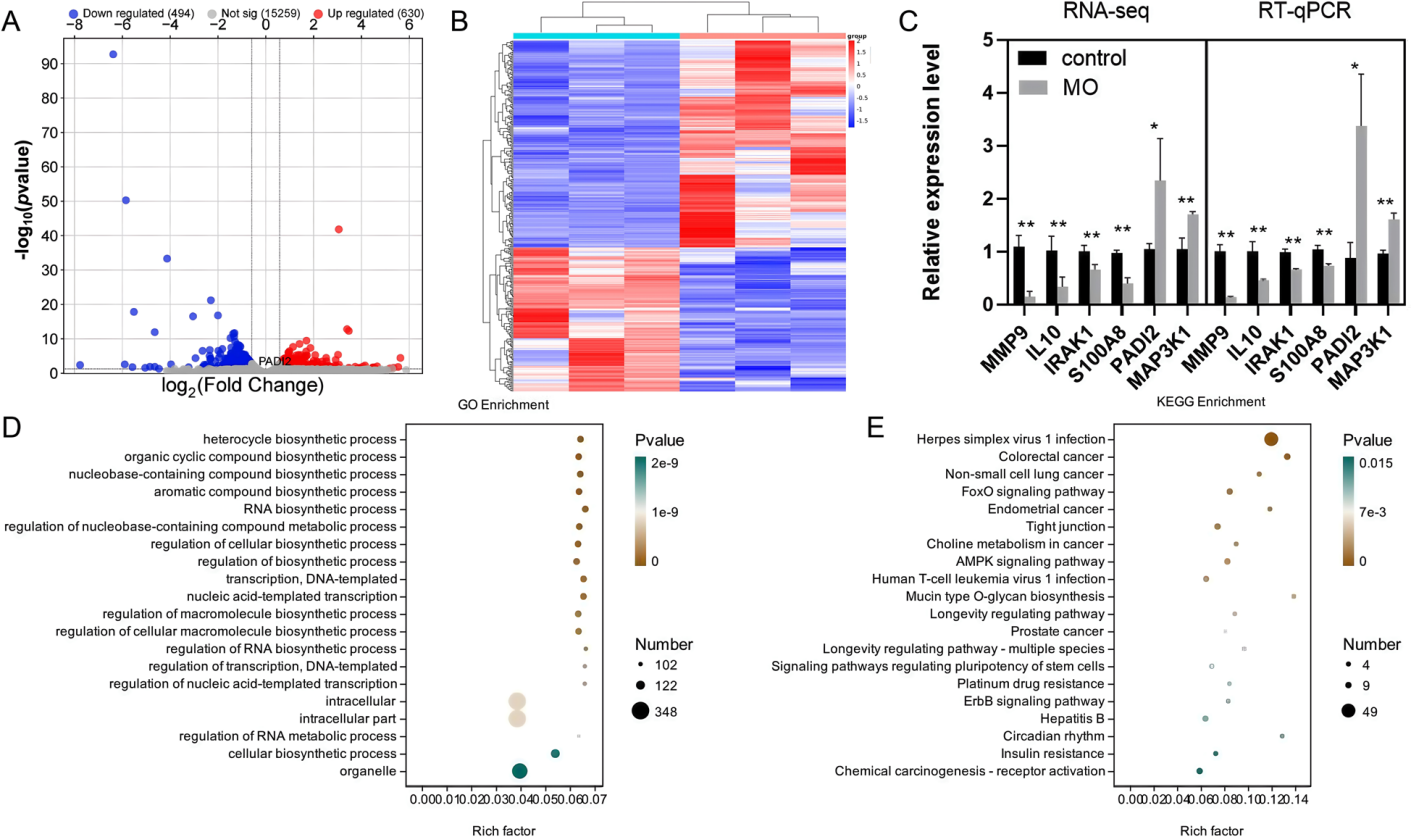
**Analysis of differentially expressed genes.** **A** Volcanic diagram of the differentially expressed genes. **B** Heatmaps of differentially expressed genes. **C** Validation of differentially expressed genes using RT‒qPCR. **D** Top 20 GO terms for up-DEGs of control-vs.-MO. **E** Top 20 KEGG pathways for upregulated DEGs in the control-vs.-MO comparison.

After 29 days of MO infection, the bronchoalveolar lavage fluid was collected, and the cells were centrifuged for transcriptome sequencing. A total of 1124 differentially expressed genes were identified, with 630 genes upregulated and 494 genes downregulated. *PADI2* expression was upregulated in the MO group (Figure 1A, Additional file 4). The heatmap shows the relationships of upregulated and downregulated genes between different groups, with Mycoplasma causing an increase in upregulated genes in alveolar lavage fluid cells (Figure 1B). Six differentially expressed genes related to tissue damage or remodelling (*MMP9*, *IL10*, *IRAK1*, *S100A8*, *PADI2*, and *MAP3K1*) were selected for RT‒qPCR validation. The RT‒qPCR validation results were consistent with the RNA-seq results (Figure 1C).

GO enrichment analysis revealed that the upregulated DEGs were enriched mainly in “RNA biosynthetic process”, “regulation of RNA biosynthetic process”, “regulation of RNA metabolic process”, etc. (Figure 1D, Additional file 5). The top 20 KEGG pathways were “Herpes simplex virus 1 infection”, “FoxO signalling pathway”, “AMPK signalling pathway”, “ErbB signalling pathway”, and others (Figure 1E, Additional file 6).

### MO increases expression of PADI2 in macrophages

**Figure 2:**
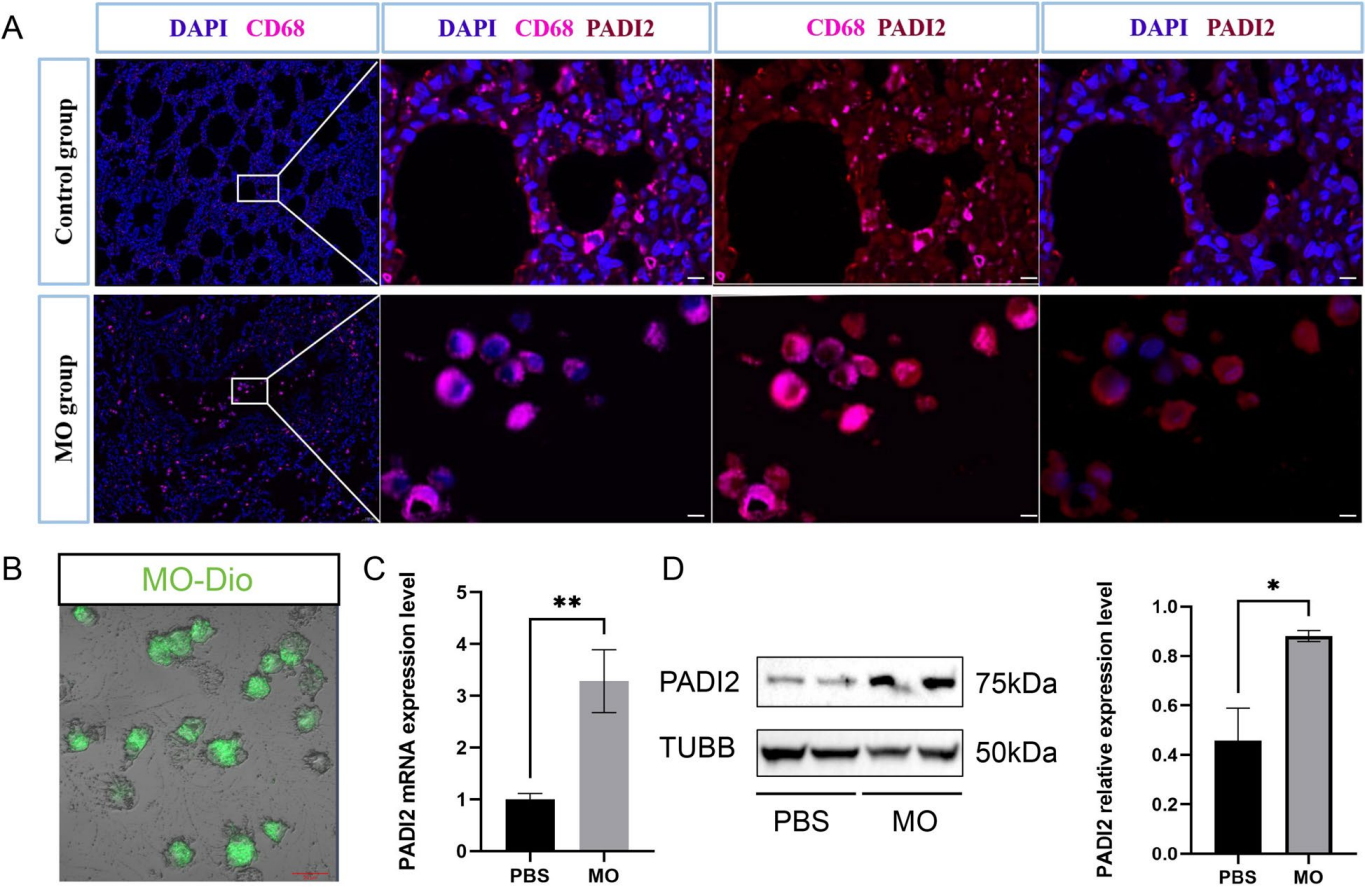
**Changes in macrophage distribution and PADI2 expression after MO infection.** **A** IHF staining of macrophages (CD68) and PADI2 in pulmonary tissue slices from the control and MO groups. Scale bar, 0.05 mm. **B** Adhesion of MO stained with DIO to BMDMs. Scale bar, 20 μm. **C** Changes in the mRNA expression of PADI2 in BMDMs after 24 h of MO infection. **D** Changes in the protein expression of PADI2 in BMDMs after 24 h of MO infection. The data are shown as the means ± SEMs from three independent experiments. *P < 0.05. **P < 0.01.

Previous research has shown that the proportion of macrophages in the lungs increases after MO infection. IHF staining of pulmonary tissue revealed a large number of free macrophages (CD68^+^) in the alveolar cavity of the MO group, whereas the macrophages in the control group were still dispersed in the alveolar wall. In addition, PADI2 was expressed in macrophages (Figure 2A). To determine the changes in the expression of PADI2 after the MO infection of macrophages, we isolated BMDMs in vitro and infected them with MO for 24 h. The results revealed that the BMDMs could be infected with DIO-stained MO (MOI = 1; Figure 2B) and that the expression level of PADI2 significantly increased (Figures 2C, D). These results indicate that MO can increase PADI2 expression in macrophages.

### Inhibition of PADI2 induces the polarization of infected macrophages towards M2 macrophages

**Figure 3:**
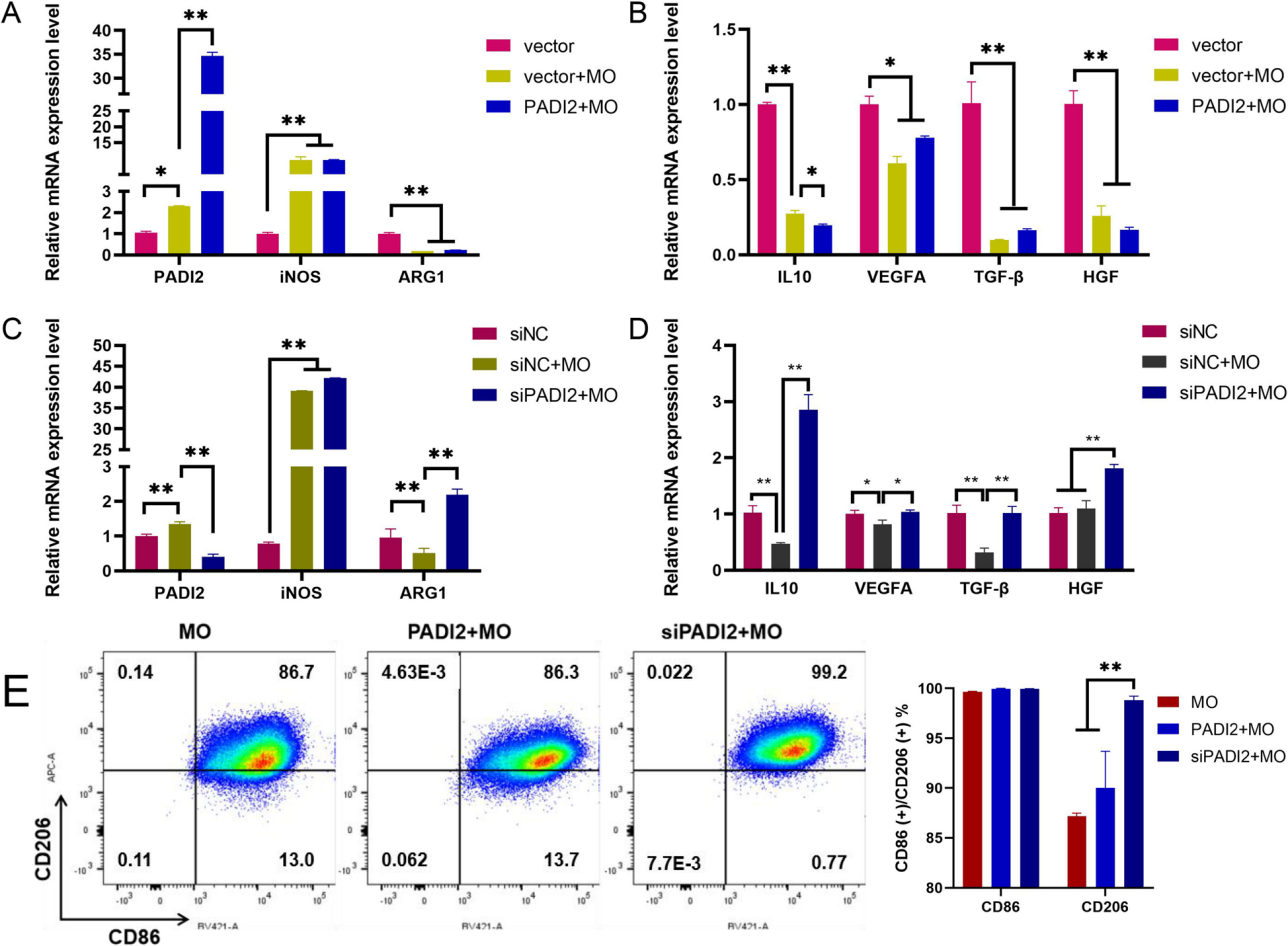
**Effect of overexpression or inhibition of PADI2 on macrophage polarization.** **A** RT‒qPCR analysis of the mRNA levels of *PADI2*, *iNOS* and *ARG1* after overexpression of PADI2. **B** RT‒qPCR analysis of the mRNA levels of IL10, VEGFA, TGF-β and HGF after overexpression of PADI2. **C** RT‒qPCR analysis of the mRNA levels of *PADI2*, *iNOS* and *ARG1* after the inhibition of PADI2. **D** RT‒qPCR analysis of the mRNA levels of IL10, VEGFA, TGF-β and HGF after the inhibition of PADI2. E: Representative results from flow cytometry analyses are shown. The data are shown as the means ± SEMs from three independent experiments. *P < 0.05. **P < 0.01.

Studies have shown that the use of PADI2 inhibitors can enhance the polarization of M2 macrophages [11]. Therefore, to investigate the function of PADI2 in infected macrophages, we designed and synthesized *PADI2* overexpression plasmids and siRNAs and transfected them into cells 6 h before mycoplasma infection. After 24 h of continuous infection, we detected the expression of *PADI2* and macrophage polarization marker genes. The results revealed that the protein levels increased or decreased after overexpression and interference, respectively, with *PADI2* (Additional file 7). The overexpression of *PADI2* increased the expression of the classically activated (CAM, M1) macrophage marker gene *iNOS*, whereas the expression of the M2 marker gene *ARG1* decreased. This finding was consistent with the trend of gene changes after simple MO infection, and the difference between the two groups was not significant (Figure 3A, *P* > 0.05). In addition, M2 macrophages can secrete the anti-inflammatory, resolving and tissue-reparative phenotype-related factors *IL-10*, *TGF-β*, *VEGFA*, and *HGF* [12]. Compared with those in the control group, the expression of *IL10*, *VEGFA*, *TGF-β*, and *HGF* decreased in the MO group and overexpression group, and the expression of *IL10* was significantly reduced in the overexpression group (Figure 3B). However, after interfering with *PADI2* expression, although *iNOS* expression also increased, *ARG1* expression significantly increased (Figure 3C), and compared with that in the MO group, the expression of *IL10*, *VEGFA*, *TGF-β*, and *HGF* increased (Figure 3D). To further verify the polarization state of the macrophages, we used flow cytometry to analyse the expression of the M1 marker CD86 and the M2 marker CD206. The results revealed that the proportion of strongly CD206-positive cells significantly increased after interference with *PADI2* expression (Figure 3E). These results indicate that overexpression of *PADI2* maintains macrophages in the M1 polarization state, whereas interference with *PADI2* expression induces the expression of M2 markers in macrophages.

### Inhibition of PADI2 alleviates the damage to lung fibroblasts caused by BMDMs under MO stimulation

**Figure 4:**
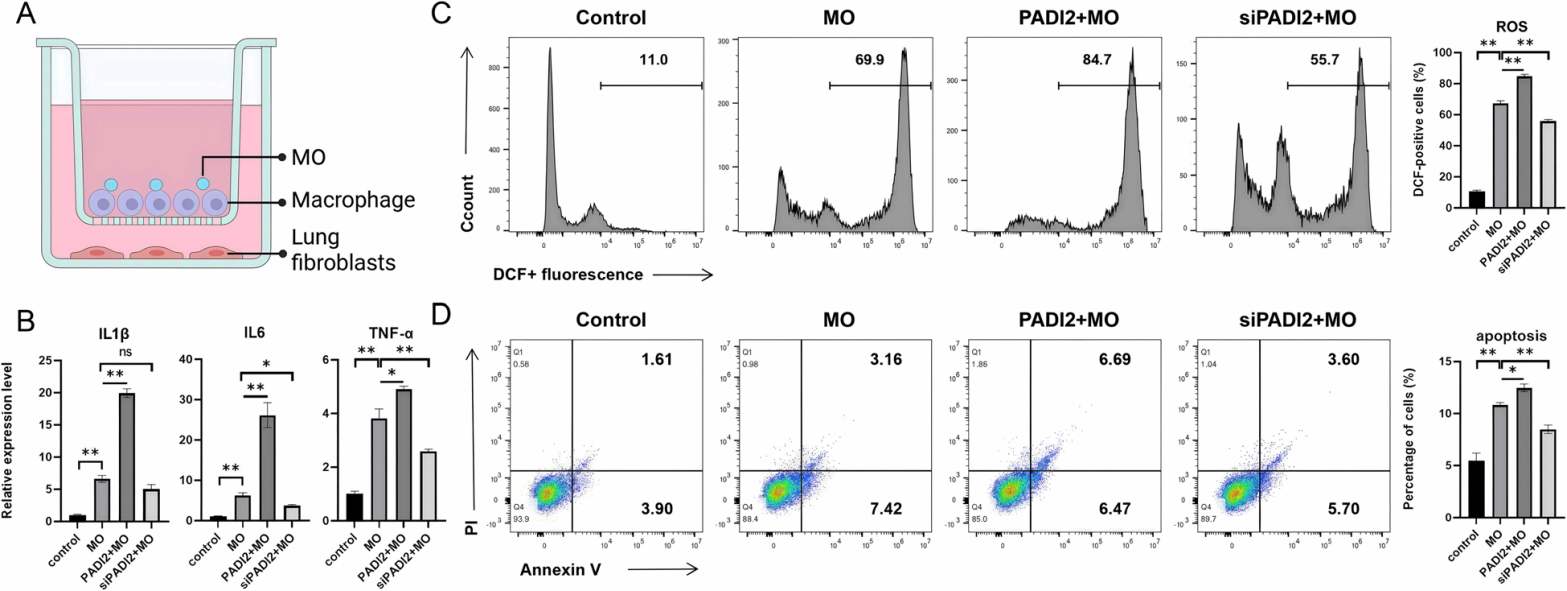
**Inhibition of PADI2 reduces apoptosis and ROS production in lung fibroblasts induced by BMDMs under MO stimulation.** **A** Schematic diagram of cell coculture. **B** RT‒qPCR detection of inflammatory factor expression in the BMDMs after 24 h of coculture. **C** The level of ROS in lung fibroblasts after 24 h of coculture was detected by flow cytometry. **D** Changes in the apoptosis of lung fibroblasts after 24 h of coculture were detected by flow cytometry. The data are shown as the means ± SEMs from three independent experiments. ^ns^*P* > 0.05. *P < 0.05. **P < 0.01.

To simulate the effect of BMDMs on lung cells in vitro, we isolated lung fibroblasts and cocultured them with BMDMs. After coculture, we first collected RNA from the BMDMs to detect the expression of inflammatory factors. The results revealed that overexpression of PADI2 promoted MO-induced expression of *IL1β*, *IL6*, and *TNF-α*, whereas inhibition of PADI2 reduced MO-induced expression of *IL6* and *TNF-α*, but the difference in *IL1β* expression was not significant (Figure 4B). We simultaneously detected apoptosis and intracellular ROS levels in fibroblasts in the coculture system. Compared with those in the MO group, both the ROS level and the apoptosis rate in the PADI2 + MO group were greater, whereas both the ROS level and the apoptosis rate were lower in the siPADI2 + MO group (Figures 4C, D). These results indicate that the overexpression of PADI2 exacerbates macrophage-induced damage to lung tissue cells, whereas the inhibition of PADI2 alleviates macrophage-induced damage to lung cells.

### The expression level of PADI2 is positively correlated with MO load

**Figure 5:**
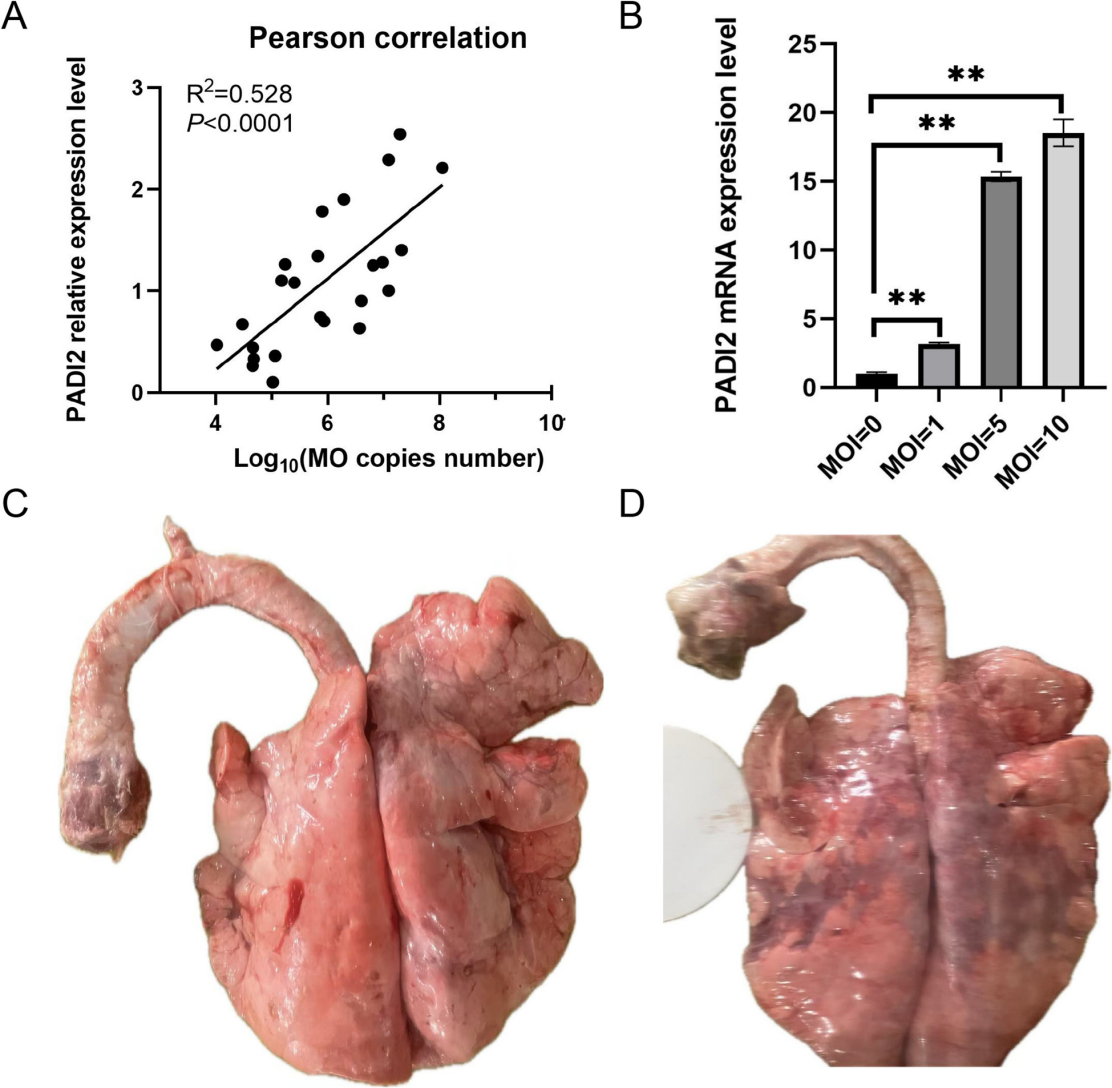
**The expression level of PADI2 is positively correlated with MO load.** **A** Analysis of the correlation between the PADI2 expression level and MO loading. **B** RT‒qPCR detection of changes in PADI2 expression in BMDMs stimulated with different MO MO MOs. **C**, **D** Pathological changes in the lungs of low-loading MO (**C**) and high-loading MO (**D**)

To determine the correlation between the mycoplasma load and PADI2 expression, we measured the endotracheal MO concentration and *PADI2* expression in the BALF of 24 diseased sheep. The results revealed that as the amount of MO in the trachea increased, the expression level of *PADI2* also increased, and there was a positive correlation between the two (Figure 5A). We stimulated BMDMs with MO at different MOIs and found that the expression level of *PADI2* increased with increasing MO concentration (Figure 5B). The pathological changes in the lungs under low and high loads of MO are shown in Figure 5C and D, respectively.

### The expression of PADI2 is regulated by the transcription factor SP3

**Figure 6:**
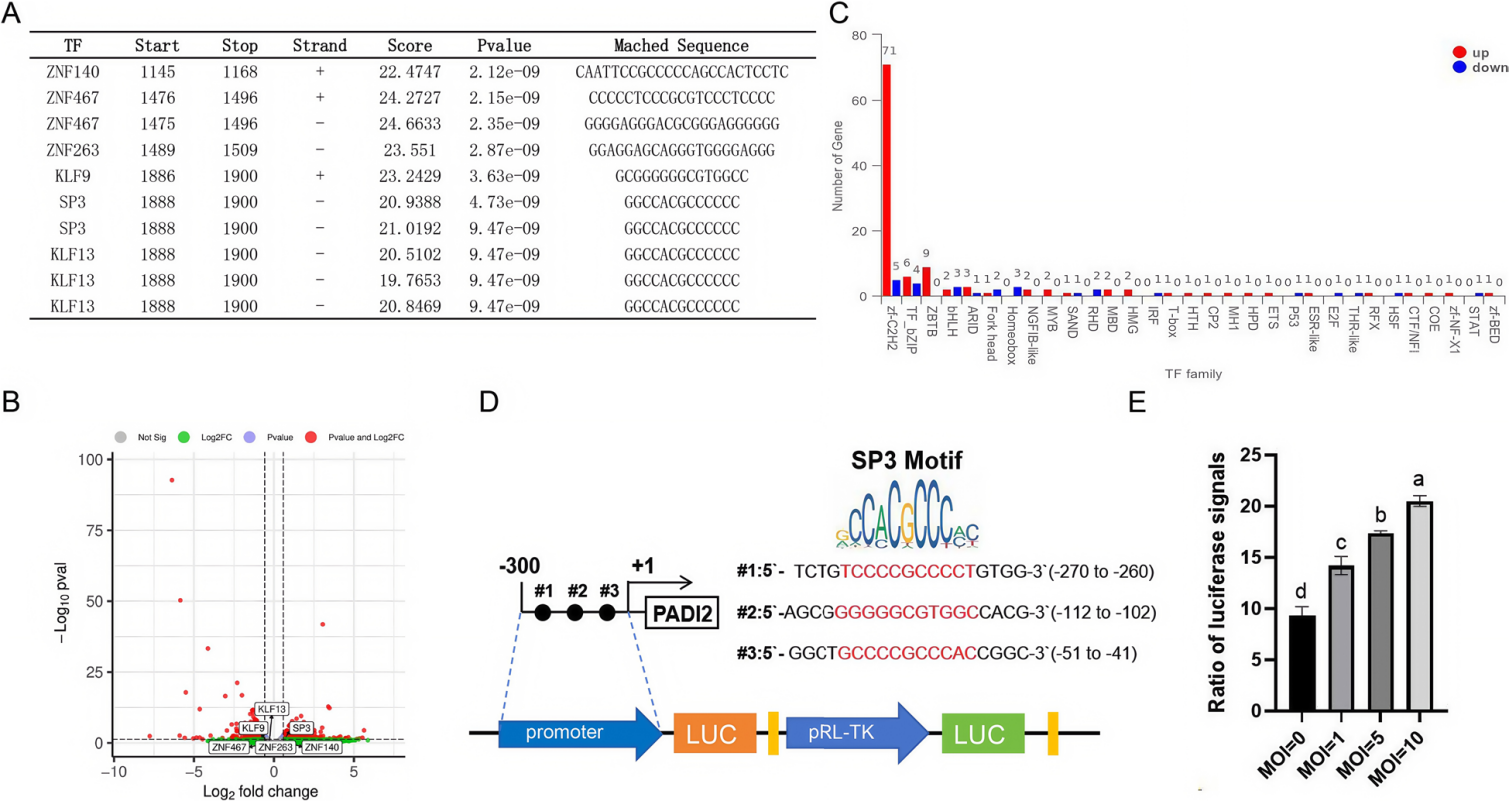
**Specific binding regions of SP3 transcription factors regulate PADI2 transcription after MO stimulation.** **A** Predicting the binding sites of the top 10 transcription factors within 2000 bp upstream of *PADI2* using the AnimalTFDB4 online website. **B** Volcano plot showing that the expression level of SP3 in the MO group was greater than that in the control group. **C** Statistical bar chart of differentially expressed transcription factor families. **D** Schematic diagram of the SP3 transcription factor-binding site 300 bp upstream of PADI2. **E** The ratio of the fluorescence signals of the BMDMs stimulated with MO at different MOIs. The data are shown as the means ± SEMs from three independent experiments. Different letters indicate significant differences between two groups.

To investigate the transcriptional regulatory mechanism underlying the upregulation of PADI2 expression after MO infection, we downloaded the sequence within 2000 bp upstream of PADI2 from the National Center for Biotechnology Information and used the transcription factor online prediction website AnimalTFDB4 v4.0 to search for transcription factors that may bind to the upstream promoter region of PADI2 (Additional file 8). Among the top ten predicted transcription factors, only SP3 was upregulated in the MO group (Figures 6A, B); SP3 belongs to the zf-C2H2 transcription factor family, whose number of upregulated transcription factors was greatest in the MO group (Figure 6C, Additional file 9). Three possible SP3 transcription factor-binding sites were predicted within 300 bp upstream of PADI2 (Figure 6D). Therefore, we constructed a dual-fluorescence reporter system, pGL3-Basic-PADI2, containing this sequence and transfected it into BMDMs, which were subsequently stimulated with different concentrations of MO. The results revealed that as the multiplicity of infection increased, the luciferase signal also increased, indicating that mycoplasma infection can increase the expression of PADI2 through the SP3 transcription factor (Figure 6E).

## Discussion

To date, several key questions regarding the response of sheep alveolar macrophages to MO infection remain unanswered. In the current study, we collected sheep alveolar macrophages that had been infected with MO for 28 days and detected changes in the expression of genes potentially involved in lung tissue damage or remodelling, such as *MMP9*, *S100A8*, *IL10*, *IRAK1*, and *MAP3K1*. These findings further reveal the potential function of PADI2 in the macrophage response to MO infection.

The main function of MMP-9 is to regulate the dynamic balance of the extracellular matrix (ECM) through degradation and remodelling [13]. During the normal wound-healing process, which involves inflammation, angiogenesis, and ECM remodelling, monocytes reach the damaged site and induce the secretion of MMP-9. During this process, monocytes become macrophages and secrete TGF-β1 and VEGF for angiogenesis, leading to ECM remodelling [14]. After MO infection, the expression of MMP9 in sheep alveolar macrophages decreased, which may affect the remodelling of lung tissue after injury. IL10 is an anti-inflammatory cytokine that can oppose the switch to the metabolic program induced by inflammatory stimuli in macrophages, promote mitochondrial autophagy, eliminate dysfunctional mitochondria characterized by low membrane potential and high levels of reactive oxygen species, and negatively regulate inflammasome activation [15]. IL10 is crucial for the polarization of M2 macrophages, and low expression of IL10 in the MO group may further inhibit M2 polarization. S100A8 and S100A9 (calprotectin) have antibacterial properties, and S100A8/A9 firmly binds to micronutrients, including zinc and manganese ions, to deprive microorganisms of these essential nutrients [16]. In addition, exposure to S100A8/A9 during the differentiation of monocytes into macrophages is beneficial for their differentiation into M2-like macrophages [17]. IRAK1 and MAP3K1 can activate the NF-κB and MAPK pathways [18, 19] and play important roles in regulating macrophage polarization [20, 21].

In this study, the expression of PADI2 increased in the BMDMs after infection. High expression of PADI2 promotes the production of IL-1β, IL-6, and TNF-α in macrophages; increases macrophage apoptosis by promoting the expression of caspase-3, caspase-2, and caspase-9; and enhances cell adhesion function [5]. Therefore, PADI2 may be an important regulatory factor for the production of inflammatory factors in infected cells. In sepsis and sepsis-induced acute lung injury, the selective inhibition of PADI2 using small-molecule inhibitors increased the survival rate of sepsis model mice. Inhibiting PADI2 expression can alleviate inflammation and increase macrophage bactericidal activity [22]. This process may be enhanced by inhibiting the ability of PADI2 to polarize M2 macrophages [11]. We also observed that inhibition of PADI2 expression enhances M2 macrophage polarization and reduces *IL6* and *TNF-α* expression, which is consistent with the findings of two recent studies. In THP-1 macrophages, inhibition of PADI2 expression can reduce the expression of TNF-α and IL-6 and increase the polarization of macrophages towards the M2 phenotype, and the NF-κB pathway is significantly enriched in this process [23]. In addition, in a PADI2 knockout mouse model, the expression of M2-related genes in macrophages is upregulated, which affects mainly the macrophage phenotype by mediating the citrullination of the nuclear factor NF-κB p65 protein, and acute lung injury in mice is improved by targeted inhibition of PADI2 [24].

Furthermore, we observed a correlation between MO loading and PADI2 expression levels and investigated the transcriptional activity of the upstream SP3 promoter-binding sequence region of PADI2 under stimulation with different concentrations of mycoplasma. Previous studies have shown that SP3 is a binding factor important for PADI2 promoter activity [25], which is consistent with our findings. The SP3 transcription factor belongs to the zf-C2H2 family, which has the greatest number of upregulated transcription factors in the MO group. Studies have shown that the zf-C2H2 transcription factor family plays important roles in animal hypoxia and acidification stress responses [26], indicating that zf-C2H2 transcription factors are widely involved in gene regulation in animal stress environments.

**Figure 7:**
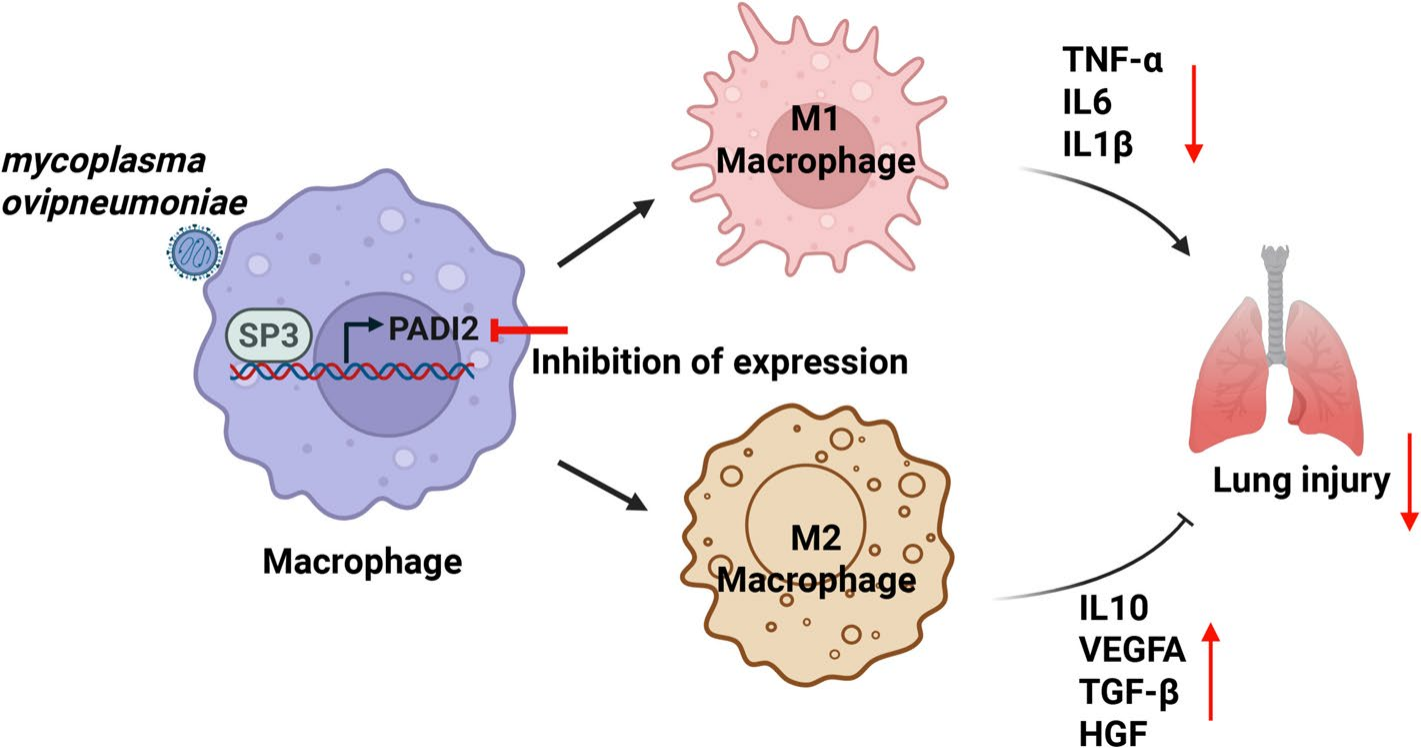
**Schematic diagram of the regulation of macrophage polarization through PADI2 after MO stimulation.** Our results indicate that after Mycoplasma infection, PADI2 transcription is upregulated through SP3 regulation, M2 polarization of macrophages is inhibited, and tissue repair-related gene expression is suppressed, thereby exacerbating lung injury.

In summary, through RNA-seq analysis, we demonstrated that sheep MO infection induces an increase in PADI2 expression in alveolar macrophages. Importantly, high expression of PADI2 may inhibit M2 polarization of macrophages, suppress lung tissue repair, and exacerbate lung injury, highlighting the important role of SP3 transcription factor-binding sites in MO-stimulated PADI2 expression (Figure 6). Our data also provide important information for future research on the function of macrophages and their response to pathogenic infections in sheep (see Figure 7).

## Supplementary Information

**Additional file 1: IF identification image of lung fibroblasts.** Vimentin expression was positive, while cytokeratin expression was negative.

**Additional file 2: siRNA sequence of PADI2.**

**Additional file 3: Primers of related genes.**

**Additional file 4: List of differently expressed genes between the MO and control groups.**

**Additional file 5: Gene Ontology (GO) enrichment analysis of upregulated differentially expressed genes**

**Additional file 6: Kyoto Encyclopedia of Genes and Genomes (KEGG) enrichment analysis of upregulated differentially expressed genes**

**Additional file 7: Expression levels of the PADI2 protein in different groups.** After overexpression or interference with PADI2 and MO infection for 24 h, the proteins extracted from cells were detected using antibodies against PADI2 and TUBB. The numerical value represents the ratio of the protein signal to the TUBB signal.

**Additional file 8: Prediction of transcription factor-binding sites upstream of PADI2.**

**Additional file 9: List of differentially expressed transcription factors between the MO and control groups.**

## Data Availability

The datasets generated during the present study are available from the NCBI under BioProject accession number PRJNA1264690.
