# Supplementary figures and images for: PADI2 exacerbates Mycoplasma ovipneumoniae-induced lung injury in sheep by suppressing M2 macrophage polarization
Source: Vet Res. 2025 Oct 27;56:203. doi: 10.1186/s13567-025-01632-7 (PMC12560429; doi:10.1186/s13567-025-01632-7)

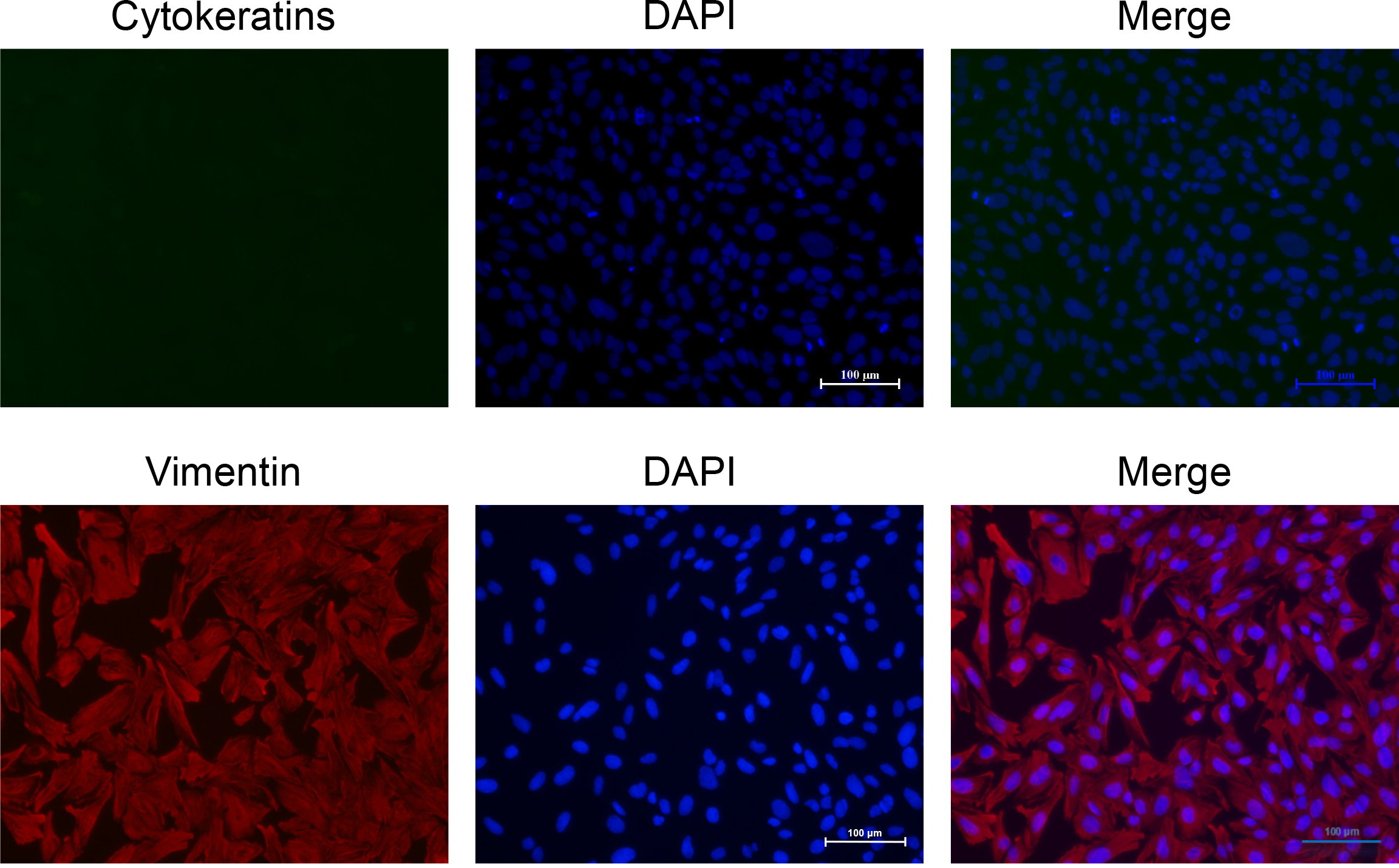

Supplement: Supplementary file 1 — Additional file 1: IF identification image of lung fibroblasts. Vimentin expression was positive, while cytokeratin expression was negative. [file 13567_2025_1632_MOESM1_ESM.tif]

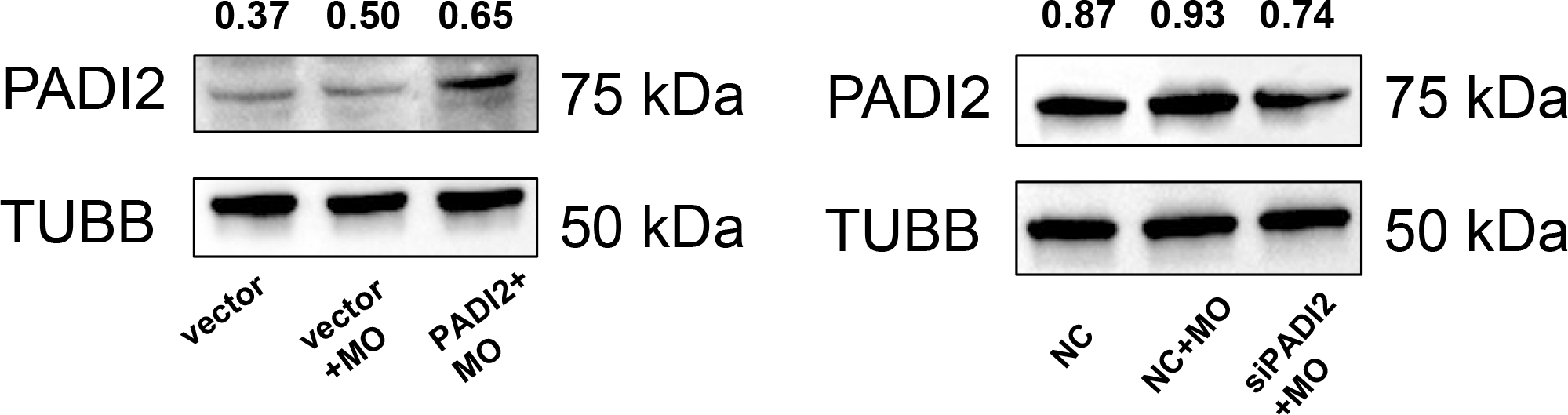

Supplement: Supplementary file 7 — Additional file 7: Expression levels of the PADI2 protein in different groups. After overexpression or interference with PADI2 and MO infection for 24 h, the proteins extracted from cells were detected using antibodies against PADI2 and TUBB. The numerical value represents the ratio of the protein signal to the TUBB signal. [file 13567_2025_1632_MOESM7_ESM.tif]
